# Drift-diffusion explains response variability and capacity for tracking objects

**DOI:** 10.1038/s41598-019-47624-4

**Published:** 2019-08-02

**Authors:** Asieh Daneshi, Hamed Azarnoush, Farzad Towhidkhah, Amin Gohari, Ali Ghazizadeh

**Affiliations:** 1 Biomedical Engineering Department, Amirkabir University of Technology (Tehran Polytechnic), Tehran, Iran; 2 Electrical Engineering Department and Brain Research Center, Sharif University of Technology, Tehran, Iran; 3 School of Cognitive Sciences, Institute for Research in Fundamental Sciences, Tehran, Iran

**Keywords:** Decision, Human behaviour

## Abstract

Being able to track objects that surround us is key for planning actions in dynamic environments. However, rigorous cognitive models for tracking of one or more objects are currently lacking. In this study, we asked human subjects to judge the time to contact (TTC) a finish line for one or two objects that became invisible shortly after moving. We showed that the pattern of subject responses had an error variance best explained by an inverse Gaussian distribution and consistent with the output of a biased drift-diffusion model. Furthermore, we demonstrated that the pattern of errors made when tracking two objects showed a level of dependence that was consistent with subjects using a single decision variable for reporting the TTC for two objects. This finding reveals a serious limitation in the capacity for tracking multiple objects resulting in error propagation between objects. Apart from explaining our own data, our approach helps interpret previous findings such as asymmetric interference when tracking multiple objects.

## Introduction

Time to contact (TTC), which is the time it takes for an object to reach an observer or a particular place, is an important factor in a variety of real-world situations, such as catching and hitting balls in games, driving vehicles, or passing through a busy street. The ability to estimate TTC for one object has been assessed in several studies (for example see^1–14^).

The accuracy and precision of TTC estimation is related to the time perception ability, which is considered in several studies^6,11,15–24^. A type of task that is often considered in laboratory studies for TTC estimation is coincidence anticipation (CA)^5^. In CA tasks, subjects must make a simple response (e.g. press a button) when the moving object reaches a particular place, called contact point^5^. In an important type of CA task, often referred to as prediction motion (PM) tasks, the moving object disappears before reaching the contact point or hides behind an occluder. Then, after a specific time, subject should indicate (often by pressing a button) the presumed time for the moving object to reach the contact point. The PM paradigm is used as a straightforward method to assess an individual’s ability to estimate absolute TTC (e.g.^4^). The main purpose of PM tasks is to understand how sensory and cognitive information are used to estimate TTC. To this aim, variables related to object’s motion, e.g., velocity, extrapolation distance and/or duration, are manipulated.

A few studies have looked at tracking multiple simultaneously moving objects. TTC judgment for two or more objects is required in a large number of everyday activities, such as crossing a multi-lane street or walking in crowded areas. One of the first studies which has considered multiple objects in TTC estimation tasks is done by Novak^25^. She presented multiple objects approaching a finish line, but the participants were asked to judge the TTC for only one of them. Most other studies presenting several objects simultaneously, have used relative judgment (RJ) tasks^3,5,26,27^. In such tasks, subjects determine which of the two (or more) moving balls arrives first at a designated goal, after disappearing^5^. Here again, participants may compute and compare several TTCs, but they are asked for only one TTC estimation. The most important difference between RJ tasks and multi-object PM tasks is that for the former, participants may misestimate TTC for some or all objects, but still give the correct answer, as long as they preserve the perceived order of arrivals. In contrast, in multi-object PM tasks, the absolute accuracy of TTC judgment is assessed.

Recently, Baures and colleagues have conducted some studies on the simultaneous estimation of the TTC for multiple objects^28–31^. However, their main focus has been on understanding how humans use their limited sources of attention to estimate several TTCs simultaneously, without considering the mechanisms underlying TTC estimation by humans.

In this study, we used one and two-object PM tasks in human subjects. Based on the data obtained, we propose a mathematical model based on drift-diffusion to explain how people estimate TTC for one object, and then extend the model for simultaneously estimation of two objects TTC.

## Materials and Methods

Eighteen students from Amirkabir University of Technology-Tehran Polytechnic (9 women, 9 men, age 26.61 years ± 3.01 (mean ± SD), min age 23, max age 33) took part in these tests voluntarily. All participants had normal or corrected-to-normal vision. They were healthy and without any known oculomotor abnormalities. All experiment protocols were approved by Iran University of Medical Sciences review board (approval ID: IR.IUMS.REC.1397.104). Participants were naive with respect to the purpose of the experiment, gave written informed consent to their participation in the experiment, and were paid for their participation. All experimental procedures were in accordance with the Declaration of Helsinki.

### Apparatus and experimental procedure

Participants sat on a chair facing a 17″ computer display located at a viewing distance of approximately 50 cm, in a silent room with normal light (1 cm corresponding to 1.14 visual degree). Stimuli were generated with MATLAB and presented on an Asus computer equipped with a 2.90 GHz Intel Corei7 processor. The screen resolution was 1920 × 1080 pixels (horizontal by vertical) and the display rate was 60 Hz.

### Experiment 1 (one-ball experiment)

In the first section (hereafter referred to as “one-ball experiment”), time-to-contact (TTC) estimates for a green ball (diameter of 1 cm) moving at constant speed on a 30 cm × 15 cm (horizontal by vertical) frontoparallel plane from left to right were obtained using a prediction motion (PM) task (see^5^). The constant speeds were randomly selected from three values: 2 cm/s, 4 cm/s, 6 cm/s. After 1.5 seconds, the ball passed behind an 18 cm × 8 cm dark grey rectangle (hereafter referred to as “occluder”) that obscured its trajectory. A 0.3 cm × 8 cm vertically-oriented red line was shown at one of five different positions on top of the occluder (at a distance of 2.5 cm, 5 cm, 7.5 cm, 10 cm, 12.5 cm from the starting point of the occluder).

**Figure 1:**
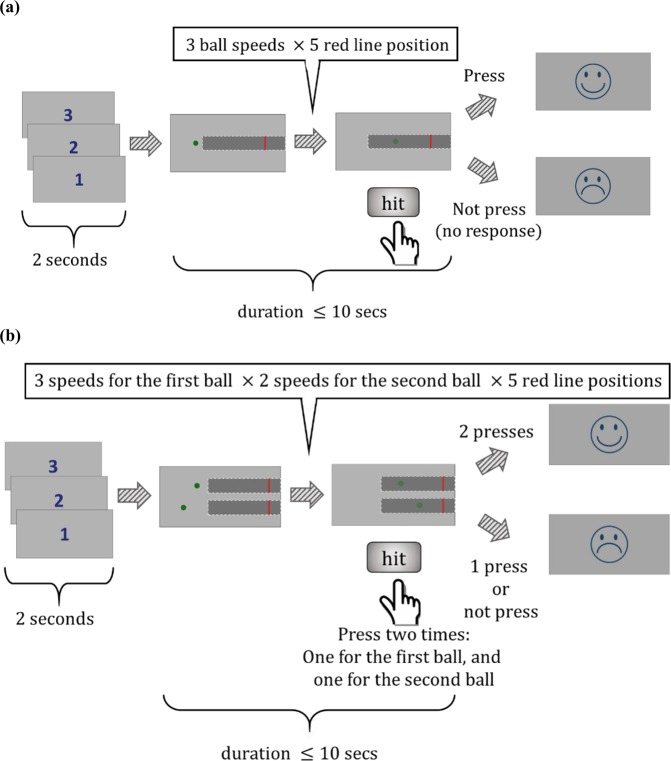
Experimental paradigm. (**a**) Schematic of one-ball experiment: after an ITI of 2 seconds, subject saw a rightward moving green ball that was presented 1.5 seconds before disappearing under an opaque cover. Subjects had to estimate the TTC for this ball with respect to the red line drawn over the cover by pressing a key after the ball went under the occluder. If the subject pressed within 10 seconds a happy emoji would be presented for 0.5 seconds, otherwise, a sad emoji was presented for 0.5 seconds. No feedback about accuracy of TTC judgement was provided. (**b**) Schematic of two-ball experiment: same procedure as the one-ball experiment except that two green balls with different speeds moving rightward were shown. Both balls went under the cover at the same time and subjects had to press the down button to estimate the TTC of each ball with respect to the red line.

Participants pressed the spacebar key to start the test. After an interatrial interval (ITI) of 2 s, a ball started to move at one of the above mentioned constant speeds, in a horizontal straight line towards the finish line. After 1.5 s, the ball passed behind the occluder and continued its motion to reach the finish line. The ball did not reappear after it was occluded. Participants had 10 seconds to press the “down” arrow key to indicate the instant at which they judged the ball would collide with the red finish line. No feedback on TTC estimation error was provided, but a smiley emoji was presented at the end of each trial if the individual finished the trial by pressing the down button and a sad emoji was presented if the subject did not press the down button in the 10-second interval and missed that trial. At the beginning of each trial we had a countdown from three to one, before the ball started moving corresponding to the 2s ITI. Figure 1(a) shows the schematics of the one-ball experiment. In this experiment, each trial condition (15 combinations of speed and finish line position) was presented 10 times in random order, for a total of 150 trials.

### Experiment 2 (two-ball experiment)

After completing the one-ball experiment, participants were tested in a second condition (hereafter referred to as “two-ball experiment”), in which two balls were presented simultaneously and moved on parallel horizontal trajectories from left to right with different velocities. The initial point for each ball was determined so that (both balls) were visible for 1.5 seconds before going behind the occluder. Therefore, both balls started moving simultaneously and reached the cover simultaneously. TTC estimates were obtained using the same method as in the one-ball experiment. Subject reported their TTC estimates by pressing the ‘down’ arrow key one time for each ball. The number of conditions in the two-ball experiment was thirty (three speeds for one of the balls, two other speeds for the other ball, and five positions for the red finish line). Each condition was presented 5 times in random order in a session, resulting in the total number of 150 trials. Figure 1(b) shows the schematics of the two-ball experiment.

### Mathematical background

We have used the following distributions for fitting TTC estimate for each subject:

1. Gaussian (normal) distribution^32^:
  1 $$f(x|\mu ,{\sigma }^{2})=\frac{1}{\sqrt{2\pi {\sigma }^{2}}}{e}^{-\frac{{(x-\mu )}^{2}}{2{\sigma }^{2}}}$$
  where *μ* is the mean or expectation of the distribution and*σ* is the standard deviation.
2. inverse Gaussian (IG) distribution^32^:
  2 $$f(x;\mu ,\lambda )={[\frac{\lambda }{2\pi {x}^{3}}]}^{\frac{1}{2}}\exp \{\frac{-\lambda {(x-\mu )}^{2}}{2{\mu }^{2}x}\}$$
  for *x* > 0. where *μ* > 0 is the mean and *λ* > 0 is the shape parameter.
3. Gamma distribution^32^:
  3 $$f(x;\alpha ,\beta )=\frac{{\beta }^{a}{x}^{\alpha -1}{e}^{-\beta x}}{{\rm{\Gamma }}(\alpha )}$$
  for *x* > 0 and *α*, *β* > 0, where Γ(*α*) is the complete Gamma function.
4. Weibull distribution^32^:
  4 $$f(x;\lambda ,k)=\{\begin{array}{cc}\frac{k}{\lambda }{(\frac{x}{\lambda })}^{k-1}{e}^{-{(\frac{x}{\lambda })}^{k}} & x\ge 0\\ 0 & x < 0\end{array}$$
  where *k* > 0 is the shape parameter and *λ *> 0 is the scale parameter of the distribution.
5. ex-Gaussian distribution^33^:

5 $$f(x;\mu ,\sigma ,\lambda )=\frac{\lambda }{2}{e}^{\frac{\lambda }{2}(2\mu +\lambda {\sigma }^{2}-2x)}erfc(\frac{\mu +\lambda {\sigma }^{2}-x}{\sqrt{2}\sigma })$$

where *erfc* is the complementary error function defined as:

6 $$erfc(x)=1-{\rm{erf}}(x)=\frac{2}{\sqrt{\pi }}{\int }_{x}^{\infty }{e}^{-{t}^{2}}dt$$

The “dfittool” function in “MATLAB” was used to fit the desired distributions to data and to get a likelihood and an R^2^ as measures of goodness of each fit.

**Figure 2:**
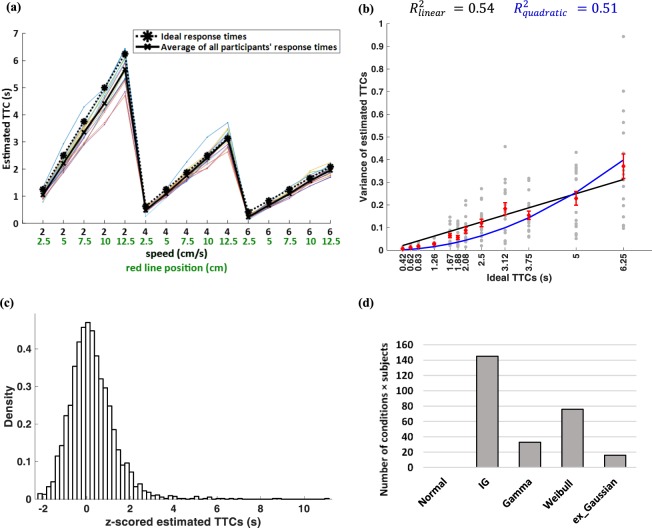
TTC estimates and error patterns in one-ball experiment. (**a**) Estimated TTCs for one-ball experiment. Each marker on the horizontal axis represents one condition composed of a given ball speed and a given red line position: (ball speed (cm/s), red line position (cm)), and the vertical axis shows the estimated TTCs averaged on similar trials. The dashed black plot shows the ideal TTC, and the thick black line shows the average of all subjects’ responses (subject’s estimated TTC). The coloured plots show the averaged response times across conditions (each condition was repeated 10 times randomly interleaved) for each participant. (**b**) Estimated TTC variability versus ideal TTCs for each subject in the one-ball experiment. The grey points represent data for each subject in a given condition. (outliers removed by procedure described in method section). The red bars show mean of each condition across subjects ± SEM. The linear and quadratic fits to the data are shown with black and blue lines, respectively. (Number of dots = conditions × participants = 15 × 18, outliers = 11) (**c**) Aggregate density of z-scored estimated TTCs for all subjects in all trials (**d**) Number of conditions × subjects in which each of the five probability distributions namely Gaussian, inverse Gaussian, Gamma, Weibull and ex-Gaussian provided the best fit according to the AIC criterion.

Plotting the subject data in Figs 2(b), 4(c) and 6(a–d) and model fitting was done after removal of outlier data points that were three scaled median absolute deviations (MAD) away from the median^34,35^.

## Results

Figure 1(a,b) shows the schematics of the one-ball and two-ball experiments. First, response times (RTs) of the one-ball experiment were studied to find out their general trend. The response times for trials belonging to the same condition were averaged (each condition was repeated 10 times randomly during the session) in the one-ball experiment and plotted in different colours for each participant in Fig. 2(a).

Results in Fig. 2(a) show that the subjects’ estimated TTCs are close to the ideal TTCs, but on average, subjects tend to slightly underestimate TTCs (thick black line below dashed black line).

In addition, for each speed, increasing the distance of the finish line from the start point, resulted in an increase in the variability of the responses between subjects (more spread in coloured lines). In order to better study response variability, the variances of response times are plotted in Fig. 2(b). Figure 2(b), shows that the TTC estimate variability increases monotonically for longer ideal TTCs. Indeed, this monotonic increase can be fit in a linear fashion (Fig. 2(b) black line, R^2^ = 0.54). Given the evidence about scaling of standard deviation (SD) of responding with duration in temporal production tasks^36,37^ and to be able to judge whether variance or SD scales better with TTC, we also fit a quadratic function of TTC to variance (i.e. Var ~ TTC^2^). The quadratic function fit was slightly worse than the linear fit in terms of R^2^ thus variance seem to be better scaled with ideal TTC compared to SD (Fig. 2(b) blue line, R^2^ = 0.51). Furthermore, we used a more general function Var ~ αTTC^n^ for fitting variance data to find the best power ‘n’. Results show the best n = 1.18 which was not found to be different from 1 but significantly different from 2 when using ideal TTC (10^5^ bootstrap p = 0.13 for comparison versus 1 and p < 0.01 for comparison versus 2).

As mentioned previously, participants tended to underestimate the TTCs (Fig. 2(a)). Therefore, we hypothesized that if the variability in TTC is to increase linearly with time, this relationship should be stronger if one considers the variability as a function of subjects estimated TTCs rather than the ideal TTCs. Indeed, the linear fit between TTC variability and estimated TTC resulted in a modest increase in the goodness of fit when using estimated TTC (R^2^ = 0.55). Again, the quadratic fit was worse than the linear fit (R^2^ = 0.52) arguing for scaling of variance rather than SD by the TTC times. The best power fit in this case (Var ~ αTTC^n^) was n = 1.25 which was closer to 1 than 2. (10^5^ bootstrap p = 0.23 for comparison versus 1 and p < 0.01 for comparison versus 2).

To further characterize response variability, we looked at the probability density function of response times. Figure 2(c) shows the density of response times in all conditions and for all participants. For this figure, in each condition we collected all the data for all the participants. Then we normalized estimated TTCs by z-scoring the data (mean value of zero and variance of one). It can be seen that the distribution of estimated TTCs is rightward-skewed.

To determine the distribution underlying the response variability, the estimated TTCs in each condition for every participant (10 similar trials are considered as one condition) were fit separately to each of the candidate distribution functions (see methods). The best distribution fit to the data from each condition and each participant was found using the AIC method (each condition is a combination of the speed of the ball and the position of the red line). Next, the number of conditions for which a given probability distribution was the best fit (had the smallest AIC) were counted across all conditions and all participants (Fig. 2(d)). As can be seen inverse Gaussian distribution provided a better fit to the data in an overwhelming majority of conditions and subjects.

**Figure 3:**
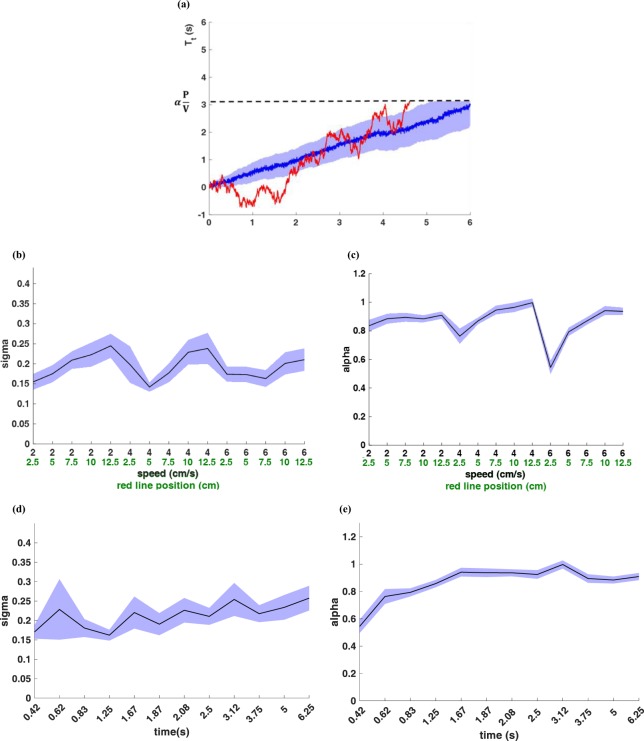
A biased drift-diffusion model for making TTC estimates. (**a**) Schematic of the drift-diffusion model for tracking one object. Red trace indicates the decision variable in a given trial, blue plot indicates the average of decision variables in a 100 trials, and the blue shade around it is the variance of this plot. The horizontal dashed line shows the decision threshold. When the decision variable reaches this threshold, a decision will be made. (**b**) *σ* values for all trials in the one-ball experiment sorted based on different trial conditions. (**c**) *α* values for all trials in the one-ball experiment sorted based on different trial conditions. (**d**) *σ* values for all trials in the one-ball experiment sorted based on ideal TTCs. (**e**) *α* values for all trials in the one-ball experiment sorted based on ideal TTCs.

In our study, subjects had to make a decision about when the ball reached the red line. One can assume that subjects can track the ball using a noisy internal timer that has to reach a bound corresponding to the ratio of the red finish line to the ball speed. Such a formulation is reminiscent of a drift-diffusion model (DDM) with a slope one drift. Although DDM has been widely used to model evidence accumulation, there is nothing intrinsic in its assumptions that limits its use outside this scope. Indeed, the DDM model has been used in interval timing tasks with the exact same logic as ours that the subjects must be using an internal clock with accumulated noise to reproduce their internally presented target times^38–44^ (also see reviews^45,46^).” These facts together with the point that the density of estimated TTCs is best fit by an inverse Gaussian, and the scaling of variances with increasing the TTC, led us to propose the DDM as the cognitive process underlying the TTC estimation in our human subjects. Furthermore, DDM has been used previously to model decisions in temporal production tasks (e.g.^41^). Figure 3(a) shows the schematic of this model for one object. In this model, we assume that subjects track passage of time after each small time step Δt (similar to a pacemaker accumulator model)^17,42,47–49^ with an additive Gaussian noise at each time step. Such a system can be modelled with a stochastic process with the following formulation:

7 $$X(t)=t+\sigma W(t)$$

where X(t) indicates the subject’s estimated time (decision variable) with the decision threshold being *P*/*v*, where *P* is the position of the finish line, and *v* is the velocity of the ball. The right side of equation 7 is composed of two terms: a constant drift *t*, that is the actual time, and the diffusion term *σW*(*t*), which represents Wiener noise drawn from a Gaussian distribution with increasing variance of *σ*^2^*t* over time representing the effect of noise accumulation. At each time point, *X*(*t*) is normally distributed with probability density *p*(*X*(*t*)) = *N*(*t*, *σ*^2^*t*)^50^. However, it can be shown that the time to hit a decision threshold in such a drift-diffusion model is rightward-skewed and follows an inverse Gaussian distribution^51^.

Therefore, drift-diffusion model could be a good candidate for TTC estimation. For one-ball experiment we have:

8 $$\begin{array}{c}T={\rm{\inf }}\{t > 0|X(t)=\frac{P}{v}\} \sim IG(\frac{P}{v},\frac{{P}^{2}}{{v}^{2}{\sigma }^{2}})=IG(\mu ,\lambda )\\ E(T)=\frac{P}{v}\,{var}(T)=\frac{P}{v}{\sigma }^{2}\end{array}$$

where *T* indicates the time to hit the threshold (first passage time), and *IG* indicates the inverse Gaussian distribution. *P* is the position of the finish line, and *v* is the speed of the ball.

Thus the integration noise *σ* can be obtained from the expected value and variance of the estimated TTC:

9 $$\sigma =\sqrt[2]{\frac{{var}(T)}{E(T)}}$$

As mentioned above (see Fig. 2(a)), participants tend to underestimate the TTCs. To account for this observation, we assume that subjects use a scaled decision threshold such that:

10 $$\alpha =\frac{E(T)}{P/v}$$

Thus, the full stochastic process will have the following formulation:

11 $$\begin{array}{c}T={\rm{\inf }}\{t > 0|X(t)=\alpha \frac{P}{v}\} \sim IG(\alpha \frac{P}{v},\frac{{\alpha }^{2}{P}^{2}}{{v}^{2}{\sigma }^{2}})\\ E(T)=\frac{\alpha P}{\upsilon }\,\,{var}(T)=\frac{\alpha P}{\upsilon }{\sigma }^{2}\end{array}$$

Figure 3(b–e) show the fitted values of *σ* and *α* for the one-ball experiment for each condition (each condition is a combination of the speed of the ball and the position of the red line) across all subjects. Results show that the effect of condition on both *σ* and *α* was significant (*F*(14,269) = 2.032, *p* = 0.016 for *σ*, and *F*(14,269) = 20.532, *p* < 0.001 for *α*). The integration noise seemed to increase with TTC and underestimation seemed lower at higher TTCs (Fig. 3(d–e). Furthermore, values of *σ* and *α* were significantly different across subjects (*F*(17,268) = 8.153, *p* < 0.001 for *σ*, and *F*(17,268) = 1.691, *p* = 0.045 for *α*).

**Figure 4:**
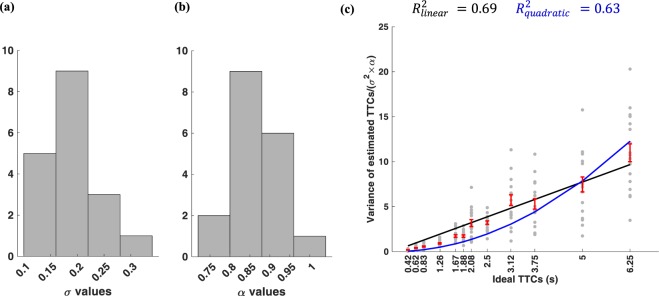
(**a**) The histogram of *σ* values for all participants in the one-ball experiment. (**b**) The histogram of *α* values for all participants in the one-ball experiment. (**c**) Variance of estimated TTCs for the one-ball experiment divided by *σ*^2^*α* versus ideal TTCs for each subject in the one-ball experiment. The format is the same as Fig. 2b.

Figure 4(a,b) shows the distribution of *σ* and *α* in TTC estimation across subjects. As noted previously, the scaling of TTC variance with TTC mean depends on value of *σ* and *α* for each subject (equation 11). Given the heterogeneity of the *σ* and *α* across subjects, a better way of probing the scaling of TTC variance by TTC mean across subjects is obtained if one normalizes the TTC variance of each subject by his/her own value of *σ* and *α*. As can be seen in Fig. 4(c), accounting for inter-subject heterogeneity improved the R^2^ of model fits with the linear model still providing a better fit to the data.

**Figure 5:**
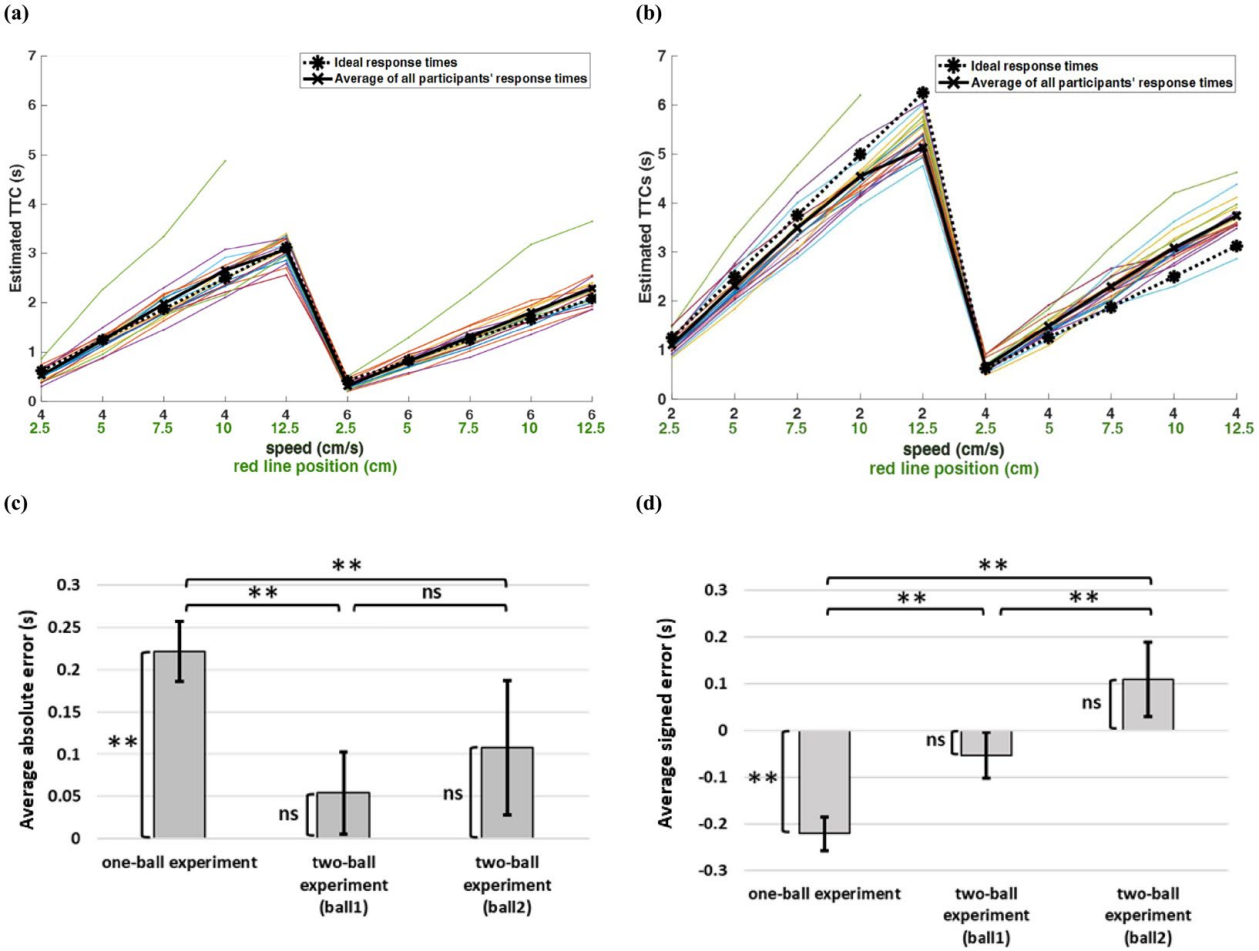
TTC estimates and error patterns in the two-ball experiment (**a**) Estimated TTCs for the first ball and (**b**) for the second ball in the two-ball experiment. Format is the same as Fig. 2a. (**c**) Absolute average TTC error for one-ball and two-ball experiments. Error bars throughout the paper show the standard error of mean (SEM). (**d**) Average signed TTC error for one-ball and two-ball experiments.

Next, to see whether subjects congnitive model using drift-diffusion in one-ball experiment can extend to cases with more balls, we repeated similar analyses in the two-ball experiment (Fig. 1(b)). Figure 5(a,b) shows the estimated TTCs of the first ball and the second ball in the two-ball experiment. Once again, subjects’ estimated TTC tended to be close to the ideal TTCs. However, there was a small tendency to overestimate TTC of the second ball with the speed of 4 cm/s and to underestimate it with the speed of 2 cm/s (ideal TTCs versus average of subjects’ estimated TTCs). Figure 5(c,d) shows the mean of TTC error across all trials and all participants for the one-ball experiment and the first ball and the second ball in the two-ball experiment. While the error in TTC estimation was in general low, the absolute error seemed to be larger in the one-ball experiment (*F*(2,8099) = 275.426, *p* < 0.001, post hoc between one-ball experiment and the first ball *p* < 0.001 and the second ball *p* = 0.006). The absolute error was not significantly different between the first and second ball (*p* = 0.015). However, the signed average TTC error was significantly different between the first and second ball in the two-balls experiment (Fig. 5(d), *F*(2,8097) = 166.283, *p* < 0.001 between three groups (one-ball experiment, and the first ball and the second ball in the two-ball experiment), also post hoc tests showed significant difference between each pair of three mentioned groups (p < 0.001)). The signed average TTC error for the one-ball condition (mean = −0.222, SD = 0.152) were significantly different from zero (t(17) = 6.165, p < 0.001). However, they were not significantly different from zero for the first ball (mean = −0.054, SD = 0.296), in the two-ball experiment (t(17) = −0.776, p = 0.448); and for the second ball (mean = 0.108, SD = 0.338), in the two-ball experiment (t(17) = 1.352, p = 0.194).

**Figure 6:**
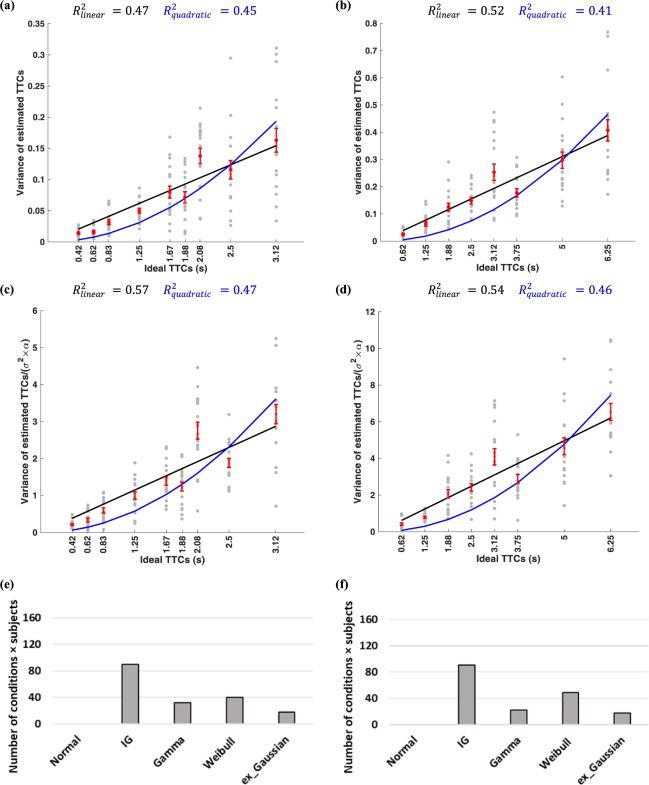
TTC distribution in the two-ball experiment (**a**) Estimated TTC variability versus ideal TTCs for each subject in the first ball experiment of the two-ball experiment. The red bars show the average of estimated TTCs for all subjects in each condition ± SEM. The outlier points (deviated more than two times the standard deviation from the mean value) are not considered. (**b**) Same as (**a**), for the second ball of the two-ball experiment. (**c**) Estimated TTC variability divided by *σ*^2^*α*, versus ideal TTCs for each subject in the first ball experiment of the two-ball experiment. (**d**) Same as (**c**), for the second ball of the two-ball experiment. In (**a**–**d**) the format is the same as Fig. 2b. (Number of dots = conditions × participants = 9 × 18 for the first ball, outliers = 10, Number of dots = conditions × participants = 8 × 18 for the second ball, outliers = 8) (**e**) Number of conditions × subjects in which each of the five probability distributions provided the best fit according to the AIC criterion for the first ball in the two-ball condition. (**f**) the same plot as (**e**) for the second ball in the two-ball condition.

In the two-ball experiment, the distribution of estimated TTCs again showed features consistent with an underlying drift-diffusion process, that is: 1) Fig. 6(a,b) show that the TTC estimate variability increases monotonically for longer ideal TTCs (black lines in Fig. 6(a) *R*^2^ = 0.47 for first ball, and Fig. 6(b) *R*^2^ = 0.52 for second ball). The normalized TTC variance (by each subjects *σ* and *α*) scales linearly with ideal TTCs (black lines in Fig. 6(c) *R*^2^ = 0.57 for first ball, and Fig. 6(d) *R*^2^ = 0.54 for second ball). Similar to the one ball experiment, this increase was better explained when using estimated compared to ideal TTCs (*R*^2^ = 0.62 for first ball, *R*^2^ = 0.63 for second ball) and 2) the shape of estimated TTC distribution was best described in majority of conditions across all subjects with inverse Gaussian for both the first and second ball (Fig. 6(e,f)). As can be seen in Fig. 6(a–d) linear fit had a slightly larger *R*^2^ compared to quadratic fit, once again suggesting variance scaling was slightly better than SD scaling in TTC estimate distribution.

Given the task, an alternate plausible model for estimating TTC could have been via a two-stage system that work in series. First stage is the TTC estimation which can have a Gaussian distribution around the correct TTC. Second stage involves the actual reproduction of the estimated TTC, which can follow a Weber like distribution with the standard deviation being scaled by estimated TTC (e.g. an exponential distribution). Such a system results in an ex-Gaussian distribution of errors (convolution of a Gaussian pdf with an exponential pdf). As can be seen in Figs 2(d) and 6(e,f) while this distribution fits some of the data, overall in both one-ball and two-ball experiments it underperforms other distributions such as Weibull and Inverse Gaussian.

**Figure 7:**
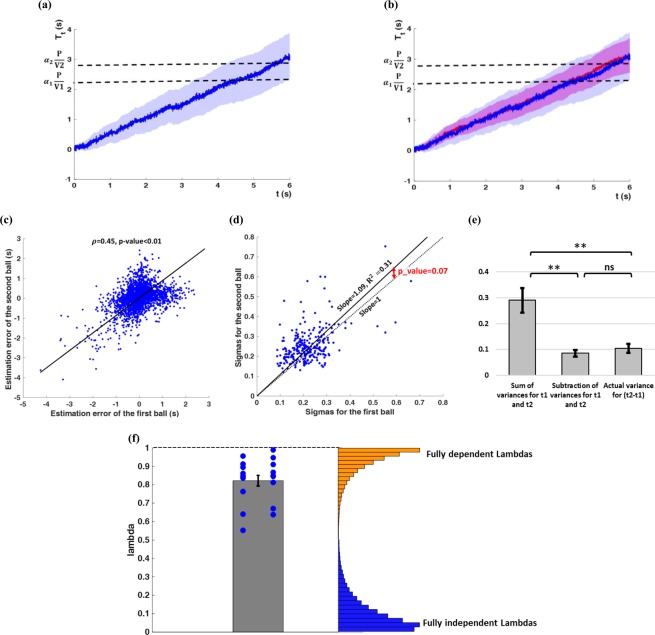
Two alternative drift-diffusion models can be used for TTC of two balls. (**a**) Schematic of the TTC model, when the participant considers a single decision variable for both balls. Here, the participant estimates the TTC of the first ball when the decision variable hits the first decision threshold and TTC of the second ball when the same decision variable now hits the second decision threshold. (**b**) Schematic of the drift-diffusion model for two objects, when the participant considers a separate decision variable for each ball. Here, the participant estimates TTC of the first ball when the first decision variable hits the first decision threshold and TTC of the second ball when the second decision variable hits the second decision threshold. (**c**) TTC estimation errors for the second ball versus estimation errors for the first ball. (**d**) *σ* values for the second ball versus *σ* values for the first ball in the two-ball experiment. Each dot belongs to one participant in a given condition. Number of dots = conditions × participants = 15 × 18. (**e**) The variance of difference in TTC estimate for ball one and ball two in two ball experiment is plotted along with the sum of variances and subtraction of variances of two TTC estimates. (**f**) Fitted *λ* values for each subject in the two-ball experiment. Each blue dot shows the average of *λ*s for one participants in all fifteen conditions (n = 18). The blue histograms at the right part of the panel show the distribution of fitted *λ* values for a 100,000 simulated samples of two completely dependent DDMs (upper histogram), and for a 100,000 simulated samples of two completely independent DDMs (lower histogram).

Theoretically, tracking objects in the two-ball experiment could be done either with a single decision variable or with two decision variables that evolve independent from each other (Fig. 7(a,b)). The formulation of drift-diffusion process in the case of single DV is:

12 $$\begin{array}{ccc}X(t) & = & t+\sigma W(t)\\ {T}_{1} & = & {\rm{\inf }}\{t > 0|X(t)=\frac{{\alpha }_{1}P}{{v}_{1}}\} \sim IG(\frac{{\alpha }_{1}P}{{v}_{1}},\frac{{{\alpha }_{1}}^{2}{P}^{2}}{{v}_{1}^{2}{\sigma }^{2}})\\ E({T}_{1}) & = & \frac{{\alpha }_{1}P}{{v}_{1}}\,{var}({T}_{1})=\frac{{\alpha }_{1}P}{{v}_{1}}{\sigma }^{2}\\ {T}_{2} & = & {\rm{\inf }}\{t > 0|X(t)=\frac{{\alpha }_{2}P}{{v}_{2}}\} \sim IG(\frac{{\alpha }_{2}P}{{v}_{2}},\frac{{{\alpha }_{2}}^{2}{P}^{2}}{{v}_{2}^{2}{\sigma }^{2}})\\ E({T}_{2}) & = & \frac{{\alpha }_{2}P}{{v}_{2}}\,{var}({T}_{2})=\frac{{\alpha }_{2}P}{{v}_{2}}{\sigma }^{2}\end{array}$$

where X(t) describes the drift-diffusion model for both balls, but the threshold to make a decision for each ball is different. The threshold for the first ball is $\frac{{\alpha }_{1}P}{{v}_{1}}$ and for the second ball $\frac{{\alpha }_{2}P}{{v}_{2}}$, where *v*_1_ and *v*_2_ are respectively the speed of the first ball and the second ball. And *P* represents the position of the red finish line for both balls. *IG* indicates the inverse Gaussian distribution.

In the case of two independent DVs one can write:

13 $$\begin{array}{c}{X}_{1}(t)=t+{\sigma }_{1}{W}_{1}(t)\\ {X}_{2}(t)=t+{\sigma }_{2}{W}_{2}(t)\\ {{\rm{T}}}_{1}={\rm{\inf }}\{t > 0|{X}_{1}(t)=\frac{{\alpha }_{1}P}{{v}_{1}}\} \sim IG(\frac{{\alpha }_{1}P}{{v}_{1}},\frac{{{\alpha }_{1}}^{2}{P}^{2}}{{v}_{1}^{2}{{\sigma }_{1}}^{2}})\\ E({T}_{1})=\frac{{\alpha }_{1}P}{{v}_{1}}\,{var}({T}_{1})=\frac{{\alpha }_{1}P}{{v}_{1}}{{\sigma }_{1}}^{2}\\ {T}_{2}={\rm{\inf }}\{t > 0|{X}_{2}(t)=\frac{{\alpha }_{2}P}{{v}_{2}}\} \sim IG(\frac{{\alpha }_{2}P}{{v}_{2}},\frac{{{\alpha }_{2}}^{2}{P}^{2}}{{v}_{2}^{2}{{\sigma }_{2}}^{2}})\\ E({T}_{2})=\frac{{\alpha }_{2}P}{{v}_{2}}\,{var}({T}_{2})=\frac{{\alpha }_{2}P}{{v}_{2}}{{\sigma }_{2}}^{2}\end{array}$$

where *X*_1_(*t*) and *X*_2_(*t*) indicate two independent the drift-diffusion models for the first ball and the second ball, respectively. *W*_1_(*t*) and *W*_2_(*t*) are independent Wiener processes.

To know whether a single or double process was used by our subjects, one can look at the relationship between the errors of estimated TTCs between the two balls. The prediction is that if subjects used a single DV, the error in the estimated TTC of the first and second ball would be correlated. That is if one was to underestimate the first ball he/she would be more likely to underestimate the second ball and vice versa. But in the two DV condition, the errors would be uncorrelated. Figure 7(c) shows that the estimation error of the second ball versus the first ball in the two-ball experiment are highly correlated. The slope of the line fitted to the data is 0.88, and the correlation between the error values of the second ball versus the error values of the first ball is 0.45.

Also, the use of a single DV for tracking both balls predicts that the integration noise *σ* for tracking the first ball and second ball in a given trial should be the same. While we cannot estimate the *σ* in one trial we can estimate it in a given condition (given ball speeds and end line position). Figure 7(d) shows *σ* to the second ball versus the first ball in the two-ball experiment. The slope of the line regressed to these point is was not significantly different from unity as expected from a single DV model (slope = 1.09, 10^6^ bootstrap SD = 0.22, t(999999) = 1.83, p = 0.07).

If one forms the variance of subtracted estimated TTCs for the first and second ball in the single DV condition, one would have:

14 $$Var({t}_{2}-{t}_{1})=abs(var({t}_{1})-var({t}_{2}))$$

and in the two independent DVs condition, one would have:

15 $$Var({t}_{2}-{t}_{1})={var}({t}_{1})+{var}({t}_{2})$$

Figure 7(e) shows the values of actual variance of the t_1_ − t_2_, and the subtraction and sum of t_1_ and t_2_ variances for all participants, and all trials. These values are calculated for a given condition in the two ball experiment (given speed of the first and second balls and redline position) the variance of subjects estimated TTCs for each ball and the variance of the difference between the two estimated TTCs for each condition. Then, the sum and subtraction of variances were formed for that condition and subject. Next, these three values (the sum and subtraction of variances and the variance of subtracted TTCs) were averaged across all conditions × subjects (15 × 18) and shown in Fig. 7(e). Interestingly, results show that the variance of subtraction is significantly different from the sum of variances (*F*(2,53) = 14.039, *p* < 0.001, post hoc *p* < 0.001) and instead is very close to subtraction of variances (post hoc *p* = 0.66). This means that on average participants tended to use a single DV for both balls in the two-ball experiment.

To do a more formal analysis of the dependence between two drift diffusion processes, we considered the following two models:

16 $$\begin{array}{c}{X}_{1}(t)=t+\sqrt[2]{\lambda }\sigma {W}_{1}(t)+\sqrt[2]{1-\lambda }\sigma {W}_{2}(t)\\ {X}_{2}(t)=t+\sqrt[2]{\lambda }\sigma {W}_{1}(t)+\sqrt[2]{1-\lambda }\sigma {W}_{3}(t)\end{array}$$

Here *X*_1_(*t*) and *X*_2_(*t*) represent two drift diffusion processes that can have arbitrary dependence in their diffusion term (λ = 0 full independence to λ = 1 full dependence, *W*_1_(*t*) is the shared noise term while *W*_2_(*t*) and *W*_3_(*t*) are the independent noise terms). Given the fact that σ for the two balls were similar for simplicity one σ was used for all noise terms. It can be seen that in this case the variance of difference in the two TTC estimates is related to the degree of dependence in the noise via the parameter λ as the following (for full derivation see Appendix I):

17 $$\mathrm{var}({T}_{1}-{T}_{2})=(1-\lambda ){\sigma }^{2}\alpha (\frac{p}{{v}_{1}}+\frac{p}{{v}_{2}})+\lambda {\sigma }^{2}{\mathbb{E}}[|{T}_{1}-{T}_{2}|]$$

using equation 17 one can estimate lambda for each subject using the sample absolute mean and sample variance for difference of TTC estimates in each condition. As can be seen in Fig. 7(f). The results show that the average lambda across all subjects is 0.82 (median = 0.85). These results are obtained by fitting lambda for each subject under the constraint that it 0 < λ < 1 (otherwise equation 16 becomes undefined). To know whether this average lambda is different from prediction of full dependence and full independence, its value was compared with a distribution of fitted lambdas from fully dependant and fully independent simulated processes. Results show that the average subject lamda not to be significantly different from prediction of fully dependant process (ranksum test, median = 0.834, p = 0.43) but is significantly different from fully independent process prediction (ranksum test, median = 0.099, p < 0.001).

**Figure 8:**
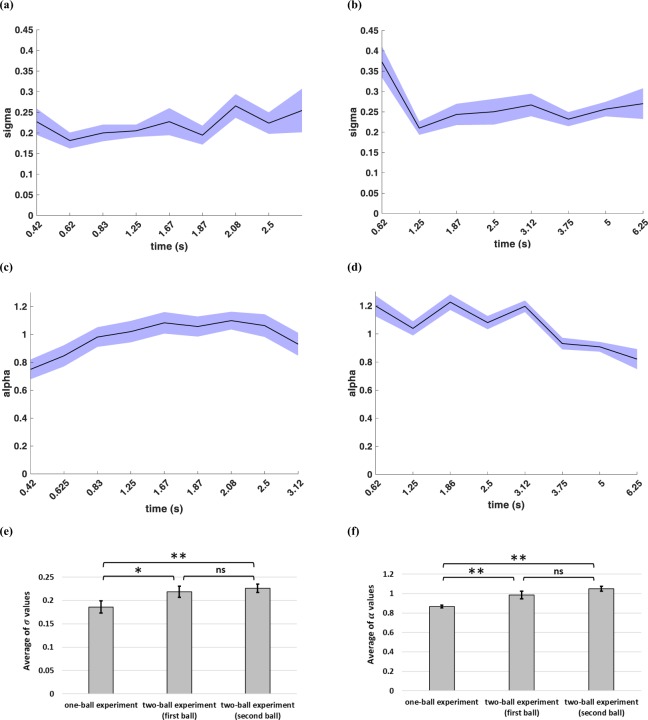
Bias and integration noise for the balls in the two ball experiment. (**a**) *σ* values for the first ball in all conditions of the two-ball experiment. (**b**) *σ* values for the second ball in all conditions of the two-ball experiment. (**c**) *α* values for the first ball in all conditions of the two-ball experiment. (**d**) *α* values for the second ball in all conditions of the two-ball experiment. (**e**) average of *σ* values, and (**f**) average of *α* values for one-ball and two-ball experiments.

To further probe the differences for tracking the two balls in the two-ball experiment, *σ* and *α* values for the two-ball experiment are calculated and shown in Fig. 8. Figure 8(a,b) show the *σ* values for the first ball and the second ball, and Fig. 8(c,d) show the *α* values for the first ball and the second ball in the two-ball experiment. Results show that the effect of condition on *σ* and *α* for both balls was significant (*F*(8,161) = 1.746, *p* = 0.092 for *σ* of the first ball, and *F*(8,161) = 5.080, *p* < 0.001 for *α* of the first ball and *F*(7,143) = 2.142, *p* = 0.043 for *σ* of the first ball, and *F*(7,143) = 5.798, *p* < 0.001 for *α* of the second ball).

Figure 8(e,f) show the average of *σ* and *α* values for all participants and all trials in one-ball experiment and two-ball experiment. ANOVA for *σ* values showed that there was a significant difference between *σ* values (*F*(2,53) = 5.132, *p* = 0.009, post hoc one-ball with first ball *p* = 0.026 and second ball *p* = 0.003). But, the difference between the first ball and the second ball in the two-ball experiment was not significant (*p* = 0. 439). Also, ANOVA for *α* values showed that there was a significant difference between *α* values (*F*(2,53) = 10.615, *p* < 0.001, post hoc one-ball with first ball *p* = 0.005 and second ball *p* < 0.001). Again the difference between the first ball and the second ball in the two-ball experiment was not significant (*p* = 0.105). The fact that that *σ* and *α* values for the first and second ball were similar on average is consistent with the fact that a single DV was used to track both balls.

## Discussion

We aimed to construct a cognitive model for human subjects tracking objects in a prediction motion task. We asked human subjects to estimate TTC of a ball hitting a finish line after it disappeared from sight. They had to make a decision about when the ball reached the finish line. Our results based on pattern of TTC estimates showed that variance of TTC error scaled with absolute TTC estimate and followed a rightward-skewed distribution that was best fit by an inverse Gaussian distribution (Fig. 2(c,d)). One natural way for estimating TTC is by an internal timer that has some added noise that is accumulated in each time step. Such formulation is reminiscent of a drift-diffusion process with a slope one drift (Fig. 3(a)). The over/underestimation of TTC was accounted for in the model by adjusting the decision bound (bias term in equation 11). Results show that the integration noise for the decision variable was relatively stable across speeds and end line positions but showed some dependence on the total TTC (Fig. 3(b,d)). When tracking two balls simultaneously subjects’ average performance was not much worse compared to one-ball condition with the bias and integration noise being comparable in the one-ball and two-ball condition (Figs 3(d,e) and 8). The actual TTC error was somewhat better in two ball experiment (Fig. 5(c,d)). This result is consistent with previous reports of good performance in concurrent interval timing in animal models^52,53^.

Interestingly, analysis of pattern of TTC errors for the two-ball experiment showed that subjects were using a single decision variable for simultaneous tracking of two balls, resulting in dependence between TTC estimates for the first and the second ball (Fig. 7(c)). This finding suggests that the errors in estimating the TTC for the first ball can easily propagate to cause miscalculation of the second ball TTCs. Our finding thus explains previous results on how errors on leading objects can affect errors for trailing objects^11,28,29,31^. It should be mentioned that in our two-ball experiment both balls went behind the occluder at the same time. Whether the same strategy can be used for cases where two balls are to disappear at different times, or to other studies of concurrent interval timing with or without onset asynchrony^52^ needs to be tested in future experiments. Ultimately, we believe that the decision to use a single process or multiple processes should be guided by cost-benefit consideration by the organism, that is if the task at hand is too important and if there is free capacity, it may be beneficial to use multiple independent processes and incur higher mental cost. This is an intriguing possibility which remains to be tested in the future.

In the one-ball experiment, increasing the ideal TTC caused *α* values to increase, saturating at about *α* = 1 for longer durations (Fig. 3(e)). This indicates that on average subjects tended to respond faster than they should in short but not long TTCs in the one-ball experiment. While the reason for this dependence is not clear at the moment this may be related to subjects’ effort not to miss short TTCs. Such underestimation of time is also seen for the faster ball with the closest end line positions as well (Fig. 8(c)). Although trends of changes in alpha between the two balls were not always easy to interpret (Fig. 8(d)). Also, increasing the ideal TTCs caused a slight increment in the *σ* values in both one-ball and two-ball experiments (Figs 3(d) and 8(a,b)). These changes in *σ* values, although small, may indicate higher order effects that are not sufficiently modeled by a simple biased drift-diffusion model.

Our finding indicates that subjects can be surprisingly accurate on average in their TTC estimates in both one and two object tracking conditions (Figs 2(a) and 5(a,b)) despite the fact that we never provided feedback about the accuracy of TTC estimates to our subjects. We found a slight overall underestimation of TTC times in one-ball experiment (but not necessarily in two-ball experiment). This result is somewhat different from some of the previous literature which found larger average errors in TTC^28,29,31^ (but see other studies which reported small errors consistent with our results^54–57^). While there is evidence that suggests speed discrimination threshold to plateau after 0.2s of viewing time^58,59^, it is possible that longer viewing time in our task compared to some previous tasks (1.5s versus 0.8s in^28^ and^29^) improved performance for example by supporting a better memory of ball speeds during the occluded period. Our subjects might have been further helped by the fact that the time under occluder was comparable to the visible time before the occlude a factor that is shown to facilitate temporal estimation^7,60^.

Some studies have shown that TTC estimates for each condition tends to be biased toward the mean TTC across all conditions^4,6,7,61^. We did not observe such pattern in our experiments. One likely reason for this difference could be due to lack of feedback in our experiment. Such feedback can result in forming priors for the subjects centred around the mean TTC which would bias estimates toward the mean in each trial. Furthermore, the overall underestimation of TTCs similar to ours have been reported previously^8,55^.

Our result show that mean of TTC estimates correlated slightly better with the variance rather than standard deviation (SD) of TTC (Figs 2(b), 4(c) and 6(a,b)). This is in contrast to broadly accepted scalar property in interval timing experiments with roots in Weber-Fechner law that suggests the SD to scale with the interval duration^62,63^. It is likely that this difference be due to the fact that tracking objects are supported by different mechanism from other time production experiments. Although, there are some evidence that challenge the scalar property in time estimation in humans and monkeys^52,64^.

In summary, we provided a rigorous cognitive model for object tracking based on a biased drift-diffusion model. Our model can predict the pattern of TTC errors and can provide a mechanism for explaining the dependence of errors when tracking two objects simultaneously. The fact that two objects were tracked with a single decision variable can show a serious limitation in working memory capacity for tracking two objects. Such a limitation can have catastrophic consequences for human observers in dealing with their environment for instance when trying to estimate TTC of incoming vehicles as miscalculation for one object can adversely affect TTC estimate for other objects. Future experiments should address whether this limitation generalizes to other configurations and occlusion asynchronies and with more objects to track. Furthermore, the neural basis for tracking objects and its overlap or dissociation from areas involved in pure time-lapse estimate needs to be studied.

## Supplementary information

Appendix I
