# Supplementary material for: Drift-diffusion explains response variability and capacity for tracking objects
Source: Sci Rep. 2019 Aug 2;9:11224. doi: 10.1038/s41598-019-47624-4 (PMC6677806; doi:10.1038/s41598-019-47624-4)
Supplement: Supplementary file 1 — Appendix I [file 41598_2019_47624_MOESM1_ESM.pdf]

Appendix I: Drift-diffusion explains response variability and capacity for tracking objects

Asieh Daneshi, Hamed Azarnoush, Farzad Towhidkhah, Amin Gohari, Ali Ghazizadeh

Let $W_1(t), W_2(t)$ and $W_3(t)$ be three independent one-dimensional standard Brownian motion processes satisfying $W_i(0) = 0$ for $i = 1, 2, 3$. The processes $X_1(t)$ and $X_2(t)$ for $t \geq 0$ are defined as follows: Let $X_1(0) = X_2(0) = 0$ and

$$X_1(t) = t + \sqrt{\lambda}\sigma W_1(t) + \sqrt{1-\lambda}\sigma W_2(t) \quad (1)$$

$$X_2(t) = t + \sqrt{\lambda}\sigma W_1(t) + \sqrt{1-\lambda}\sigma W_3(t). \quad (2)$$

Let $d_1 = P/v_1$, $d_2 = P/v_2$ and

$$T_1 = \inf\{t \geq 0 : X_1(t) = d_1\}, \quad (3)$$

$$T_2 = \inf\{t \geq 0 : X_2(t) = d_2\}. \quad (4)$$

In other words, T_1 and T_2 are the first hitting times for the two processes X_1 and X_2 respectively.

Theorem 1. *We have*

$$\mathsf{Var}(T_1 - T_2) = (1-\lambda)\sigma^2(d_1 + d_2) + \lambda\sigma^2\mathbb{E}\big[|T_1 - T_2|\big].$$

Remark 2. *Using Jensen's inequality for the function $|x|$ we can write $\mathbb{E}\big[|T_1 - T_2|\big] \geq |\mathbb{E}\big[T_1 - T_2\big]| = |d_1 - d_2|$. Therefore,*

$$\mathsf{Var}(T_1 - T_2) \geq (1-\lambda)\sigma^2(d_1 + d_2) + \lambda\sigma^2|d_1 - d_2|.$$

Proof. Let

$$D_T = T_1 - T_2.$$

and

$$\Delta T = |T_1 - T_2|.$$

We are interested in the variance of D_T. We have

$$\mathbb{E}[D_T] = \mathbb{E}[T_1] - \mathbb{E}[T_2] = d_1 - d_2.$$

Furthermore,

$$\mathbb{E}[D_T^2] = \mathbb{E}[\Delta T^2].$$

Then, to compute the variance of D_T, one should compute

$$\mathsf{Var}(D_T) = \mathbb{E}[\Delta T^2] - (d_1 - d_2)^2.$$

Let $T = \min(T_1, T_2)$. Then

$$T_1 + T_2 = 2T + \Delta T.$$

Taking expectations on both sides, we obtain

$$\mathbb{E}[\Delta T] = d_1 + d_2 - 2\mathbb{E}[T]. \tag{5}$$

Consider two events $\mathcal{E}_1$ and $\mathcal{E}_2$ as follows: $\mathcal{E}_1$ is the event that $X_1(t)$ hits the boundary d_1 first and $\mathcal{E}_2 = \mathcal{E}_1^c$ is the event that $X_2(t)$ hits the boundary d_2 first. For $\mathbb{E}[\Delta T^2]$, we have

$$\mathbb{E}[\Delta T^2] = p(\mathcal{E}_1)\mathbb{E}[\Delta T^2|\mathcal{E}_1] + p(\mathcal{E}_2)\mathbb{E}[\Delta T^2|\mathcal{E}_2]. \tag{6}$$

Assume that $\mathcal{E}_1$ occurs. Then consider the conditional distribution of X_2 at time T_1. If $X_2 = x_2 < d_2$, then the extra time that it takes to hit d_2 has an inverse Gaussian distribution with parameters $IG(d_2 - x_2, \frac{(d_2-x_2)^2}{\sigma^2})$. Thus, given $X_2 = x_2 < d_2$ at time T_1, the mean of ΔT is equal to $d_2 - x_2$ and its variance is $(d_2 - x_2)\sigma^2$; expected value of ΔT^2 would be $(d_2 - x_2)\sigma^2 + (d_2 - x_2)^2$. Using the law of iterated expectations, we can now write

$$\begin{aligned}
\mathbb{E}[\Delta T|\mathcal{E}_1] &= \mathbb{E}\left[\mathbb{E}_{X_2(T_1)|\mathcal{E}_1}[\Delta T|\mathcal{E}_1, X_2(T_1)]\right] = \mathbb{E}\left[d_2 - X_2(T_1)|\mathcal{E}_1\right], &(7)\\
\mathbb{E}[\Delta T^2|\mathcal{E}_1] &= \mathbb{E}\left[\mathbb{E}_{X_2(T_1)|\mathcal{E}_1}[\Delta T^2|\mathcal{E}_1, X_2(T_1)]\right] = \mathbb{E}\left[(d_2 - X_2(T_1))\sigma^2 + (d_2 - X_2(T_1))^2|\mathcal{E}_1\right] &(8)\\
&= \sigma^2\mathbb{E}[\Delta T|\mathcal{E}_1] + \mathbb{E}\left[(d_2 - X_2(T_1))^2|\mathcal{E}_1\right] &(9)
\end{aligned}$$

Similarly,

$$\mathbb{E}[\Delta T^2|\mathcal{E}_2] = \sigma^2\mathbb{E}[\Delta T|\mathcal{E}_2] + \mathbb{E}\left[(d_1 - X_1(T_2))^2|\mathcal{E}_2\right]. \tag{10}$$

Then, from (6), we obtain

$$\mathbb{E}[\Delta T^2] = \sigma^2\mathbb{E}[\Delta T] + p(\mathcal{E}_1)\mathbb{E}\left[(d_2 - X_2(T_1))^2|\mathcal{E}_1\right] + p(\mathcal{E}_2)\mathbb{E}\left[(d_1 - X_1(T_2))^2|\mathcal{E}_2\right]. \tag{11}$$

It remains to compute these quantities.

Fix some $a_1, a_2 \in \mathbb{R}$. Observe that

$$a_1X_1(t) + a_2X_2(t) = (a_1 + a_2)t + \alpha_1W_1(t) + \alpha_2W_2(t) + \alpha_3W_3(t),$$

where

$$\begin{aligned}
\alpha_1 &= (a_1 + a_2)\sqrt{\lambda}\sigma,\\
\alpha_2 &= a_1\sqrt{1-\lambda}\sigma,\\
\alpha_3 &= a_2\sqrt{1-\lambda}\sigma.
\end{aligned}$$

Let

$$M(t) = e^{\alpha_1W_1(t) - \frac{1}{2}\alpha_1^2t} \times e^{\alpha_2W_2(t) - \frac{1}{2}\alpha_2^2t} \times e^{\alpha_3W_3(t) - \frac{1}{2}\alpha_3^2t}.$$

The terms $e^{\alpha_1W_i(t) - \frac{1}{2}\alpha_i^2t}$ for $i = 1, 2, 3$ are known as the exponential martingale in the literature [1, p.18]. Observe that $M(t)$ is also a Martingale because it is the product of three independent Martingales. We can also express $M(t)$ as follows

$$M(t) = e^{a_1X_1(t) + a_2X_2(t) + g(a_1,a_2)t}$$

where

$$g(a_1, a_2) = -a_1 - a_2 - \frac{1}{2}(a_1 + a_2)^2 \lambda \sigma^2 - \frac{1}{2}a_1^2(1-\lambda)\sigma^2 - \frac{1}{2}a_2^2(1-\lambda)\sigma^2.$$

Observe that $M(t) \geq 0$. Furthermore, if $a_1, a_2 \geq 0$, we have $g(a_1, a_2) \leq 0$ and before the particles hitting the boundaries,

$$M(t) \leq e^{a_1 d_1 + a_2 d_2 + t \times g(a_1, a_2)} \leq e^{a_1 d_1 + a_2 d_2}.$$

Therefore $M(t)$ is bounded (before hitting the boundaries) and we can apply Martingale's optional stopping time theorem (see [1, p.22] for a similar argument in a classical setting). If $T = \min(T_1, T_2)$ is the hitting time of the first process, we have

$$\mathbb{E}M(T) = \mathbb{E}M(0) = 1.$$

Then,

$$\mathbb{E}e^{a_1 X_1(T) + a_2 X_2(T) + g(a_1, a_2)T} = 1.$$

Therefore,

$$p(\mathcal{E}_1)\mathbb{E}\left[e^{a_1 X_1(T) + a_2 X_2(T) + g(a_1, a_2)T} | \mathcal{E}_1\right] + p(\mathcal{E}_2)\mathbb{E}\left[e^{a_1 X_1(T) + a_2 X_2(T) + g(a_1, a_2)T} | \mathcal{E}_2\right] = 1.$$

In other words,

$$p(\mathcal{E}_1)\mathbb{E}\left[e^{a_1 d_1 + a_2 X_2(T_1) + g(a_1, a_2)T_1} | \mathcal{E}_1\right] + p(\mathcal{E}_2)\mathbb{E}\left[e^{a_1 X_1(T_2) + a_2 d_2 + g(a_1, a_2)T_2} | \mathcal{E}_2\right] = 1. \quad (12)$$

Let us differentiate (12) with respect to a_1. After differentiation we get

$$p(\mathcal{E}_1)\mathbb{E}\left[(d_1 + \frac{\partial g(a_1, a_2)}{\partial a_1}T_1)e^{a_1 d_1 + a_2 X_2(T_1) + g(a_1, a_2)T_1} | \mathcal{E}_1\right] \quad (13)$$

$$+ p(\mathcal{E}_2)\mathbb{E}\left[(X_1(T_2) + \frac{\partial g(a_1, a_2)}{\partial a_1}T_2)e^{a_1 X_1(T_2) + a_2 d_2 + g(a_1, a_2)T_2} | \mathcal{E}_2\right] = 0. \quad (14)$$

Setting $a_1 = a_2 = 0$, we obtain

$$p(\mathcal{E}_1)\mathbb{E}\left[d_1 - T_1 | \mathcal{E}_1\right] + p(\mathcal{E}_2)\mathbb{E}\left[X_1(T_2) - T_2 | \mathcal{E}_2\right] = 0. \quad (15)$$

Observing that

$$p(\mathcal{E}_1)\mathbb{E}\left[T_1 | \mathcal{E}_1\right] + p(\mathcal{E}_2)\mathbb{E}\left[T_2 | \mathcal{E}_2\right] = \mathbb{E}[T], \quad (16)$$

where $T = \min(T_1, T_2)$, we can write the above as

$$p(\mathcal{E}_1)d_1 + p(\mathcal{E}_2)\mathbb{E}\left[X_1(T_2) | \mathcal{E}_2\right] = \mathbb{E}[T]. \quad (17)$$

Similarly,

$$p(\mathcal{E}_2)d_2 + p(\mathcal{E}_1)\mathbb{E}\left[X_2(T_1) | \mathcal{E}_1\right] = \mathbb{E}[T]. \quad (18)$$

Next, differentiation of (12) twice with respect to a_1 gives us

$$p(\mathcal{E}_1)\mathbb{E}\left[\left(\frac{\partial^2 g(a_1, a_2)}{\partial^2 a_1}T_1 + \left(d_1 + \frac{\partial g(a_1, a_2)}{\partial a_1}T_1\right)^2\right) e^{a_1 d_1 + a_2 X_2(T_1) + g(a_1, a_2)T_1} | \mathcal{E}_1\right]$$

$$+ p(\mathcal{E}_2)\mathbb{E}\left[\left(\frac{\partial^2 g(a_1, a_2)}{\partial^2 a_1}T_2 + \left(X_1(T_2) + \frac{\partial g(a_1, a_2)}{\partial a_1}T_2\right)^2\right) e^{a_1 X_1(T_2) + a_2 d_2 + g(a_1, a_2)T_2} | \mathcal{E}_2\right] = 0. \quad (19)$$

Setting $a_1 = a_2 = 0$ and observing that

$$\frac{\partial g(a_1,a_2)}{\partial a_1}(0,0) = -1$$

$$\frac{\partial^2 g(a_1,a_2)}{\partial^2 a_1} = -\lambda\sigma^2 - (1-\lambda)\sigma^2 = -\sigma^2.$$

Then,

$$p(\mathcal{E}_1)\mathbb{E}\left[-\sigma^2 T_1 + (d_1 - T_1)^2 \,|\mathcal{E}_1\right] + p(\mathcal{E}_2)\mathbb{E}\left[-\sigma^2 T_2 + (X_1(T_2) - T_2)^2 \,|\mathcal{E}_2\right] = 0. \tag{20}$$

Then

$$-\sigma^2\mathbb{E}[T] + p(\mathcal{E}_1)\mathbb{E}\left[(d_1 - T_1)^2 \,|\mathcal{E}_1\right] + p(\mathcal{E}_2)\mathbb{E}\left[(X_1(T_2) - T_2)^2 \,|\mathcal{E}_2\right] = 0, \tag{21}$$

and similarly,

$$-\sigma^2\mathbb{E}[T] + p(\mathcal{E}_2)\mathbb{E}\left[(d_2 - T_2)^2 \,|\mathcal{E}_2\right] + p(\mathcal{E}_1)\mathbb{E}\left[(X_2(T_1) - T_1)^2 \,|\mathcal{E}_1\right] = 0. \tag{22}$$

Next, let us differentiate (12) with respect to a_1 and a_2. After differentiation we get

$$\begin{aligned} &p(\mathcal{E}_1)\mathbb{E}\left[\left(\frac{\partial^2 g(a_1,a_2)}{\partial a_1 \partial a_2}T_1 + \left(d_1 + \frac{\partial g(a_1,a_2)}{\partial a_1}T_1\right)\left(X_2(T_1) + \frac{\partial g(a_1,a_2)}{\partial a_2}T_1\right)\right)e^{a_1 d_1 + a_2 X_2(T_1) + g(a_1,a_2)T_1}|\mathcal{E}_1\right] \\ &+ p(\mathcal{E}_2)\mathbb{E}\left[\left(\frac{\partial^2 g(a_1,a_2)}{\partial a_1 \partial a_2}T_2 + \left(X_1(T_2) + \frac{\partial g(a_1,a_2)}{\partial a_1}T_2\right)\left(d_2 + \frac{\partial g(a_1,a_2)}{\partial a_2}T_2\right)\right)e^{a_1 X_1(T_2) + a_2 d_2 + g(a_1,a_2)T_2}|\mathcal{E}_2\right] = 0. \end{aligned} \tag{23}$$

Setting $a_1 = a_2 = 0$ and observing that

$$\frac{\partial g(a_1,a_2)}{\partial a_1}(0,0) = -1 \tag{24}$$

$$\frac{\partial^2 g(a_1,a_2)}{\partial a_1 \partial a_2} = -\lambda\sigma^2. \tag{25}$$

Then,

$$\begin{aligned} &p(\mathcal{E}_1)\mathbb{E}\left[\left(-\lambda\sigma^2 T_1 + (d_1 - T_1)\left(X_2(T_1) - T_1\right)\right)|\mathcal{E}_1\right] \\ &+ p(\mathcal{E}_2)\mathbb{E}\left[\left(-\lambda\sigma^2 T_2 + \left(X_1(T_2) - T_2\right)(d_2 - T_2)\right)|\mathcal{E}_2\right] = 0. \end{aligned} \tag{26}$$

Then

$$-\lambda\sigma^2\mathbb{E}[T] + p(\mathcal{E}_1)\mathbb{E}\left[(d_1 - T_1)\left(X_2(T_1) - T_1\right)|\mathcal{E}_1\right] + p(\mathcal{E}_2)\mathbb{E}\left[\left(X_1(T_2) - T_2\right)(d_2 - T_2)|\mathcal{E}_2\right] = 0. \tag{27}$$

Multiplying (27) by -2 and adding it up with (22) and (21), we obtain

$$2\lambda\sigma^2\mathbb{E}[T] - 2\sigma^2\mathbb{E}[T] + p(\mathcal{E}_1)\mathbb{E}\left[(d_1 - X_2(T_1))^2 \,|\mathcal{E}_1\right] + p(\mathcal{E}_2)\mathbb{E}\left[(d_2 - X_1(T_2))^2 \,|\mathcal{E}_2\right] = 0. \tag{28}$$

or

$$p(\mathcal{E}_1)\mathbb{E}\left[(d_1 - X_2(T_1))^2 \,|\mathcal{E}_1\right] + p(\mathcal{E}_2)\mathbb{E}\left[(d_2 - X_1(T_2))^2 \,|\mathcal{E}_2\right] = 2(1-\lambda)\sigma^2\mathbb{E}[T]. \tag{29}$$

We have

$$(d_1 - X_2(T_1))^2 = (d_2 - X_2(T_1))^2 + 2(d_1 - d_2)(d_2 - X_2(T_1)) + (d_1 - d_2)^2$$
$$(d_2 - X_1(T_2))^2 = (d_1 - X_1(T_2))^2 + 2(d_2 - d_1)(d_1 - X_1(T_2)) + (d_2 - d_1)^2$$

Replacing these in (29), we obtain

$$\begin{aligned} & p(\mathcal{E}_1)\mathbb{E}\left[(d_2 - X_2(T_1))^2 \,|\mathcal{E}_1\right] + p(\mathcal{E}_2)\mathbb{E}\left[(d_1 - X_1(T_2))^2 \,|\mathcal{E}_2\right] \\ &= 2(1-\lambda)\sigma^2\mathbb{E}[T] - (d_1 - d_2)^2 \\ &\quad - 2(d_1 - d_2)p(\mathcal{E}_1)\mathbb{E}\left[d_2 - X_2(T_1)|\mathcal{E}_1\right] \\ &\quad - 2(d_2 - d_1)p(\mathcal{E}_2)\mathbb{E}\left[d_1 - X_1(T_2)|\mathcal{E}_2\right] \end{aligned} \tag{30}$$

Then from (11), we have

$$\begin{aligned} & \mathbb{E}[\Delta T^2] - \sigma^2\mathbb{E}[\Delta T] \\ &= 2(1-\lambda)\sigma^2\mathbb{E}[T] - (d_1 - d_2)^2 \\ &\quad - 2(d_1 - d_2)p(\mathcal{E}_1)\mathbb{E}\left[d_2 - X_2(T_1)|\mathcal{E}_1\right] \\ &\quad - 2(d_2 - d_1)p(\mathcal{E}_2)\mathbb{E}\left[d_1 - X_1(T_2)|\mathcal{E}_2\right] \end{aligned} \tag{31}$$

Let us now compute

$$p(\mathcal{E}_1)\mathbb{E}\left[d_2 - X_2(T_1)|\mathcal{E}_1\right] - p(\mathcal{E}_2)\mathbb{E}\left[d_1 - X_1(T_2)|\mathcal{E}_2\right] \tag{32}$$
$$= p(\mathcal{E}_1)d_2 - p(\mathcal{E}_2)d_1 - p(\mathcal{E}_1)\mathbb{E}\left[X_2(T_1)|\mathcal{E}_1\right] + p(\mathcal{E}_2)\mathbb{E}\left[X_1(T_2)|\mathcal{E}_2\right] \tag{33}$$

From (17) and (18) that

$$p(\mathcal{E}_1)d_1 + p(\mathcal{E}_2)\mathbb{E}\left[X_1(T_2)|\mathcal{E}_2\right] = \mathbb{E}[T]. \tag{34}$$

and

$$p(\mathcal{E}_2)d_2 + p(\mathcal{E}_1)\mathbb{E}\left[X_2(T_1)|\mathcal{E}_1\right] = \mathbb{E}[T]. \tag{35}$$

Subtracting these two, we obtain

$$p(\mathcal{E}_2)\mathbb{E}\left[X_1(T_2)|\mathcal{E}_2\right] - p(\mathcal{E}_1)\mathbb{E}\left[X_2(T_1)|\mathcal{E}_1\right] = -p(\mathcal{E}_1)d_1 + p(\mathcal{E}_2)d_2. \tag{36}$$

Then,

$$p(\mathcal{E}_1)\mathbb{E}\left[d_2 - X_2(T_1)|\mathcal{E}_1\right] - p(\mathcal{E}_2)\mathbb{E}\left[d_1 - X_1(T_2)|\mathcal{E}_2\right] \tag{37}$$
$$= p(\mathcal{E}_1)d_2 - p(\mathcal{E}_2)d_1 - p(\mathcal{E}_1)d_1 + p(\mathcal{E}_2)d_2 \tag{38}$$
$$= d_2 - d_1. \tag{39}$$

Replacing this in (31), we obtain

$$\begin{aligned} & \mathbb{E}[\Delta T^2] - \sigma^2\mathbb{E}[\Delta T] \\ &= 2(1-\lambda)\sigma^2\mathbb{E}[T] - (d_1 - d_2)^2 \\ &\quad - 2(d_1 - d_2)(d_2 - d_1) \\ &= 2(1-\lambda)\sigma^2\mathbb{E}[T] + (d_1 - d_2)^2 \end{aligned}$$

Finally, since

$$2\mathbb{E}[T] = d_1 + d_2 - \mathbb{E}[\Delta T]. \tag{40}$$

we can write

$$\mathbb{E}[\Delta T^2] - \sigma^2\mathbb{E}[\Delta T] = (1-\lambda)\sigma^2(d_1 + d_2 - \mathbb{E}[\Delta T]) + (d_1 - d_2)^2$$

or

$$\mathbb{E}[\Delta T^2] = (1-\lambda)\sigma^2(d_1 + d_2) + (d_1 - d_2)^2 + \lambda\sigma^2\mathbb{E}[\Delta T].$$

The result follows by noting that

$$\mathsf{Var}(D_T) = \mathbb{E}[\Delta T^2] - (d_1 - d_2)^2.$$

□

Given the formulation of drift-diffusion model for the TTC task, let:

$$\begin{aligned} D_T &= T_2 - T_1, \\ \Delta T &= |T_2 - T_1|, \\ d_1 &= P/v_1, \\ d_2 &= P/v_2. \end{aligned}$$

Then,

$$\mathsf{Var}(T_1 - T_2) = (1-\lambda)\sigma^2(\frac{P}{v_1} + \frac{P}{v_2}) + \lambda\sigma^2\mathbb{E}\big[|T_1 - T_2|\big].$$

Acknowledgement

The authors acknowledge helpful discussions with Erfan Salavati.

References

[1] Rogers, L. Chris G., and David Williams. Diffusions, markov processes, and martingales: Volume 1, foundations. Vol. 1. Cambridge university press, 2000.
